# Unauthorized Version

**Published:** 1991-03

**Authors:** 


### UNAUTHORIZED VERSION

A 'mole' at the Bristol Royal Infirmary tells me that at the last Hospital Committee Meeting it was explained to the assembled consultants that under the rules of the new Special Trust, when they have fulfilled the Provider contract for the number of patients to be seen in a particular week, they will not be allowed to see any more. The following anonymous communication has been received:

God rest you merry, Gentlemen,
For unto you I say,
The District General Manager
Has promised come what may,
To save us from the Welfare State
Which led us all astray,
O tidings of comfort and joy.

The aim of the White Paper
Is very plain to see,
It's not designed to help the doctors
Such as you and me,
The main aim is to change the rules
So care's no longer free.
O tidings of comfort and joy.

When you have seen your quota
Of patients new and old,
"You must not see another one"
Is what you will be told,
And if you don't obey the rules
The Manager will scold.
O tidings of comfort and joy.

The Clinical Director, he
Will draw up your contract.
The number of procedures done
Will have to be exact.
And if you do another one
You're likely to be sacked.
O tidings of comfort and joy.

Anon

